# Shifts in Species Composition Constrain Restoration of Overgrazed Grassland Using Nitrogen Fertilization in Inner Mongolian Steppe, China

**DOI:** 10.1371/journal.pone.0016909

**Published:** 2011-03-01

**Authors:** Qing Chen, David U. Hooper, Shan Lin

**Affiliations:** 1 Department of Plant Nutrition, China Agricultural University, Beijing, People's Republic of China; 2 Department of Biology, Western Washington University, Bellingham, Washington, United States of America; University of Zurich, Switzerland

## Abstract

Long-term livestock over-grazing causes nitrogen outputs to exceed inputs in Inner Mongolia, suggesting that low levels of nitrogen fertilization could help restore grasslands degraded by overgrazing. However, the effectiveness of such an approach depends on the response of production and species composition to the interactive drivers of nitrogen and water availability. We conducted a five-year experiment manipulating precipitation (NP: natural precipitation and SWP: simulated wet year precipitation) and nitrogen (0, 25 and 50 kg N ha^-1^ yr^-1^) addition in Inner Mongolia. We hypothesized that nitrogen fertilization would increase forage production when water availability was relatively high. However, the extent to which nitrogen would co-limit production under average or below average rainfall in these grasslands was unknown.

Aboveground net primary production (ANPP) increased in response to nitrogen when precipitation was similar to or higher than the long-term average, but not when precipitation was below average. This shift in limitation was also reflected by water and nitrogen use efficiency. Belowground live biomass significantly increased with increasing water availability, but was not affected by nitrogen addition. Under natural precipitation (NP treatment), the inter-annual variation of ANPP was 3-fold greater than with stable water availability (CV_ANPP_ = 61±6% and 17±3% for NP and SWP treatment, respectively) and nitrogen addition increased CV_ANPP_ even more (89±14%). This occurred in part because fertilizer nitrogen left in the soil in dry years remained available for uptake during wet years and because of high production by unpalatable annual species in wet years in the NP treatment. In summary, plant growth by residual fertilizer nitrogen could lead to sufficient yields to offset lack of additional production in dry years. However, the utility of fertilization for restoration may be constrained by shifts in species composition and the lack of response by belowground biomass, which reduces replacement of soil carbon and nitrogen.

## Introduction

As the main grassland region of China and part of the world's largest contiguous steppe ecosystem, the Inner Mongolian steppe plays an important role in livestock farming and environmental conservation [1]. However, this ecosystem has been severely degraded in recent decades from increasing human pressures, including traditional utilization of livestock dung as fuel for cooking and heating, and poor management, such as serious overgrazing [2], [3]. Disturbance by long-term over-grazing can cause deterioration of soil chemical and physical properties in grasslands, such as decreased organic carbon and total nitrogen concentrations by at least two mechanisms [4]. First, heavy grazing often results in lower vegetation cover, accelerated soil evaporation, and altered storage of water in the soil [5], [6], thus favoring soil erosion by wind with associated loss of soil organic carbon and nitrogen [7], [8]. Second, long-term livestock over-grazing also may cause a negative imbalance in nutrient input and output, leading to soil resource depletion [3], [9], [10]. For both of these reasons, potentially greater resource limitation could occur in heavily grazed sites.

Since nitrogen (N) is an important limiting resource in Inner Mongolian steppe, applying nitrogen may be a useful approach to restore degraded grasslands [11], [12]. However, the effectiveness of nitrogen addition as a restoration technique may depend on water availability. In arid and semiarid ecosystems, precipitation is often the major primary limiting factor for plant growth and productivity [13], [14], in which case nitrogen fertilization may only be effective at increasing rangeland production in wet years. However, dryland systems often respond to nitrogen fertilization as well as increased water availability. Co-limitation by water and nitrogen could occur if each increases production independently and in combination. Alternatively, limitation could shift from water in dry years, to co-limitation in moderate precipitation years, to nitrogen in wet years [15], [16]. Furthermore, production response to precipitation can depend on rainfall regime (i.e., frequency of rainfall and amount of water per rainfall event) as much as on absolute amounts [17]–[20]. In Inner Mongolian grassland, ANPP correlated with precipitation from 1980 to 2004 with the exception of four extraordinarily wet years [21], implying that the primary limiting factors might have shifted from water to nitrogen during wet years [15], [22]. However, there has been a lack of long-term studies assessing nitrogen and water limitation in degraded grasslands in Inner Mongolia to clearly resolve this issue.

In addition to questions about primary limitation of grassland production, added nitrogen can have negative consequences for plant and soil communities. Many studies in Europe and America have found that nitrogen enrichment in native grasslands causes loss of species by favoring opportunistic annual species and/or invasion of exotics in plant communities, especially at high levels of nitrogen application [23]–[25]. Consequently added nitrogen may increase the inter-annual variation of production due to shifts in species composition under naturally variable weather conditions [26], [27]. Regions with different management regimes may respond to additional nitrogen in different ways [28]–[30] and the effects of low levels of nitrogen addition, which are most relevant to herders, remain poorly understood at heavily grazed sites in Inner Mongolian grassland.

This multi-year study investigated the effects of nutrient and water limitation on plant production in a heavily grazed site in Inner Mongolia. We used low levels of nitrogen application (25–50 kg N ha^−1^, equivalent to nitrogen deposition in other regions) and additional water in an amount to emulate the long-term mean growing season precipitation in wet years in the region. We ran the experiment for five years and analyzed the relationships of ANPP, belowground production and ecosystem resource use efficiency with precipitation and nutrient availability to test four hypotheses: 1) nitrogen co-limits production with water under natural precipitation regimes, such that addition of either would lead to increased primary production and biomass, but addition of both would give the largest response; 2) nitrogen application would increase the inter-annual variation of production across years, but this effect would depend on precipitation; 3) fertilizer use efficiency would increase with increasing precipitation and water use efficiency would increase with increasing nitrogen availability, reflecting co-limitation by water and nitrogen; and 4) important forage species (perennial grasses) would show less strong responses to nitrogen than unpalatable annual species.

## Methods

### Study site

We conducted this study at the Inner Mongolian Grassland Ecosystem Research Station (IMGERS), located in the Xilin River Basin (43°26′–44°29′N, 115°32′–117°12′E), Inner Mongolia, China. The average annual precipitation was 343 mm. More than 80% of the annual precipitation occurs between May and September, approximately covering the plant-growing season from May to late August [21] (Figure S1). From the 22 year climate database, mean annual temperature was 0.7°C and >0 average daily temperature was 12.2°C. The heavily grazed site (HG) had a stocking rate of 4 sheep ha^−1^ during the past 30 years, resulting in different plant species composition and soil physical and chemical properties compared to more moderately grazed sites [4]. The dominant plant species at the HG site were *Artemisia scoparia*, *Artemisia frigida, Carex korshinskyi* and *Potentilla tanacetifolia* at the beginning of the experiment. The predominant soil types are Calcic Chernozems derived from aeolian sediments above acid volcanic rocks [31]. No fertilizers had been applied before they were fenced for this study in May 2005.

### Fertilization and irrigation

In May 2005, an area of 0.2 ha each was fenced at the HG site, and the vegetation was mown down to 3 cm height in April 2005. The experiment was designed as a two-factorial split-plot, combined over years with 4 replicates of each treatment. The main plots were two water supply levels, i.e. natural precipitation (NP, no additional water supply) and simulated wet year precipitation (SWP), within which were subplots with three nitrogen fertilizer (urea) rates at 0, 25, and 50 kg N ha^−1^ (N0, N25, and N50). The dimensions of each subplot were 5 m ×8 m. The watering treatments and subplots were separated by 3 m and 0.8 m walkways, respectively. To apply fertilizer evenly, we mixed granular urea (1.5 mm diameter) with air-dried and fine-sieved (<2 mm) soil particles at a ratio of 1∶10 and spread this mixture by hand on May 15^th^ every year.

We determined the amount of water in the SWP treatment from the long-term rainfall data (1982–2003) obtained from the meteorological station at IMGERS. The rainfall data were ranked from driest to wettest years and divided into three groups: dry (6 years), moderate (10 years) and wet (6 years). The mean annual precipitation for dry, moderate, and wet years were 201 mm, 248 mm, and 431 mm, respectively. Supplemental irrigation was applied to simulate the amount and distribution of the long-term wet year precipitation from May to September. The fields were irrigated at 10-day intervals with the amount of the average wet year 10-day precipitation during the same period using a pump-line injector system at a windless time (often at sunset). If the actual rainfall in a given 10-day interval during the experimental period was greater than the historical wet year precipitation in the same period, irrigation was withheld for that 10-day interval and the amount of irrigation in the subsequent 10-day interval was adjusted according to the actual precipitation received in the previous 10-day interval.

### Sampling methods and data collection

We took plant tissue samples at peak aboveground biomass production on August 15–16 each year. Three aboveground plant samples were taken from 0.25 m ×1 m areas of each subplot by clipping all plant material at the soil surface. Quadrats were located to eliminate spatial overlap among all years. We composited the samples, separated plant material by species, and separated green vegetation from standing dead tissue and litter. After oven-drying (75°C for 48 hours) and weighing for dry mass, we milled the samples first in a micro hammer mill (Culatti, Zurich, Switzerland) and then with a ball mixer mill (MM200, Retsch, Haan, Germany). Nitrogen and carbon concentrations of the samples were analyzed by an elemental analyzer (EA1108, Carlo Erba, Torino, Italy). We estimated aboveground net primary production (ANPP) from the aboveground biomass of live plants.

We took plant root samples on August 17–20 each year during 2006–2009 using a 100 mm diameter soil auger down to a depth of 50 cm in the same area where plant materials were clipped. The samples were placed into mesh bags (mesh size 0.4 mm), and cleaned under a water stream. We separated root samples into live and dead root parts by a combination of magnifying glass, hand-sorting and wet sieving. We determined dead and live roots visually based on color and flexibility. Roots were subsequently oven dried at 75°C for 48 hours, and then weighed for dry mass. At the end of September each year, vegetation was mown down to 3 cm height for all treatments with clippings removed from the site for hay. This practice emulated actual management effects of grazing as closely as possible by eliminating potential effects of biomass carry-over.

We took soil samples from 0–15, 15–30 and 30–50 cm depths from all plots on 12–14 May before fertilization and 15–17 August after plant sampling during 2005–2009. For each plot, we composited three soil cores collected using a soil auger 30 mm diameter. We stored all soil samples in a refrigerator at 4°C until extraction (within 2 days) with 100 mL of 0.01 M CaCl_2_. The extracts were analyzed for ammonium (NH_4_^+^) and nitrate (NO_3_^−^) by Continuous Flow Analysis (Autoanalyzer TRAACS Model 2000, Bran and Luebbe, Germany). At same time, soil moisture was measured using the gravimetric method (48 hours at 108°C).

### Statistics and data analysis

We performed statistical analyses using SAS version 8.0 (SAS, 1996); PROC MIXED was used for analysis of variance (ANOVA) and repeated measures was used for effects of year. We tested significance of treatments according to the model for split-plot design, where year, water, and nitrogen were considered as fixed effects and the random effect was ‘*Block x water*’. Blocks had no significant effect on any measured variable and thus are not reported in the results. Multiple comparisons of means were done with the Tukey test.

We estimated water use efficiency (WUE) by the ratio of ANPP to the annual water input:
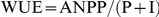
where P and I are crop year precipitation (1^st^ September of last year to 30^th^ August this year) and growing season irrigation (1^st^ May to 30^th^ August), respectively. Crop Year is the time period used for agricultural commodities, it is the duration from one year's harvest to the next, and has been used in many other studies [32], [33].

Accumulated nitrogen fertilizer use efficiency (aNUE) was assessed by:
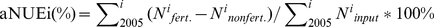
where i is year; N^i^_fert._ and N^i^_nonfert._ are the aboveground plant nitrogen content in a given year in the fertilized and non-fertilized treatments, respectively. Plant nitrogen content was determined by plant aboveground tissue nitrogen concentration times aboveground biomass. N^i^_input_ is the nitrogen fertilizer amount in a given year. We used the accumulated nitrogen efficiency because measurements indicated that substantial nitrogen remained in the soil following dry years, and this nitrogen can be used for growth in subsequent years [34], [35].

## Results

**Figure 1:**
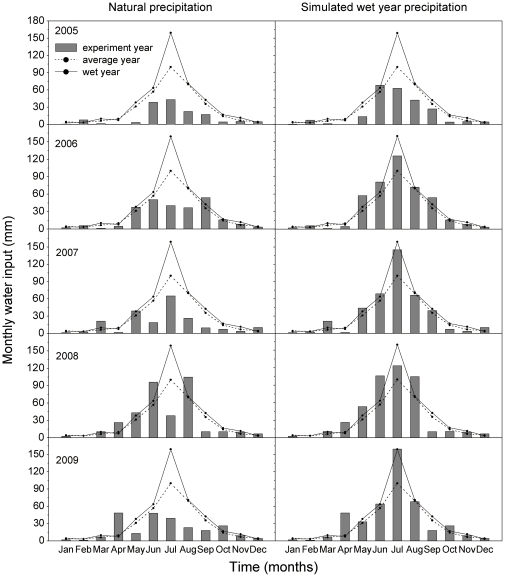
Monthly water input of long-term average and wet years and of individual experiment years 2005–2009. For the natural precipitation treatments (NP, left panel), the column represents precipitation only; for the simulated wet year precipitation treatments (SWP, right panel), the column represents precipitation plus irrigation. The solid and dotted line indicate long-term average and wet year precipitation during 1982–2003, respectively.

Under natural conditions, annual and growing season precipitation during the experimental periods were much lower than the long-term average precipitation and even lower than the mean value of dry years, with the exception of 2008 (Figure 1 left panels). However, the precipitation before the growing season in 2007 (mainly during fall and winter, September 2006– April 2007) was similar to the long-term wet year average, which led to high soil moisture in 2007 (Figure S2). Using precipitation and soil moisture, we defined 2005, 2006 and 2009 as dry years, and 2007 and 2008 as moist years in this text. The water inputs in SWP treatments agreed well with the long-term mean wet year precipitation and its patterns except in 2005 when we had technical difficulties establishing the irrigation system (Figure 1, right panels). The mean annual precipitation of the five experimental years was 243 and 398 mm in NP and SWP treatments, respectively (Figure 1).

### Primary production, root biomass and allocation

**Figure 2:**
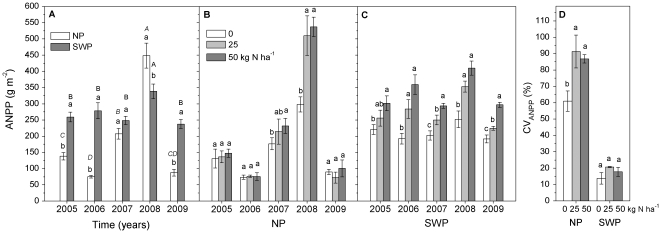
Aboveground net primary production (ANPP, g m-2 yr-1) as influenced by year, water and nitrogen. (A) ANPP as influenced by year and water interaction, with data pooled over all nitrogen treatments (n = 12). (B, C) ANPP as influenced by the nitrogen by water by year interaction (n = 4). (D) Coefficients of variation of ANPP (CVANPP%) among 5 years (n = 4). Bars labeled with the same lowercase letters were not significantly different (P>0.05) between watering treatments (A) or among nitrogen treatments (B, C). In panel A, bars labeled with the same italic and erect capital letter represent no significant difference among years in NP and SWP treatments, respectively (P>0.05).

Above ground net primary production was significantly affected by water, nitrogen, and water by nitrogen interactions, and these effects depended on year (Table 1). Looking across years, ANPP in the NP treatment was significantly higher in the moist years of 2007 and 2008 than in the dry years of 2005, 2006 and 2009, while in the SWP treatment there were no significant differences among years except for somewhat higher production in 2008 (Figure 2A). Not surprisingly, watering effects depended on amounts of natural precipitation in each year. ANPP was significantly higher in SWP than in NP in years with low precipitation (2005, 2006 and 2009; Figure 1 and 2A), while it was the same in 2007 and significantly lower in SWP than in NP in 2008 (Figure 2A).

**Table 1:** Degrees of freedom and F-statistics from repeated measures analysis to assess the effects of year (Y, from 2005–2009), water (W), and nitrogen (N) on aboveground net primary production (ANPP, g m^−2^), water use efficiency (WUE, g m^−2^ mm^−1^), accumulated nitrogen fertilizer use efficiency (aNUE, *%*), belowground live biomass (BGB_L_, g m^−2^) and the fraction of total dry mass allocated belowground (*f*_BGBL_).

Source	DF	ANPP	WUE	aNUE	DF	BGB_L_	f_BGBL_
Y	4	111.69*** §	49.78***	14.47***	3	12.09***	8.76***
W	1	42.19***	0.06ns	12.84**	1	7.80*	2.27ns
W*Y	4	48.99***	38.47***	10.9***	3	1.13ns	8.85***
N	2	37.63***	23.62***	0.14ns	2	0.23ns	10.34***
N*Y	8	5.07***	2.72*	0.59ns	5	0.51ns	0.74ns
W*N	2	3.94*	0.98ns	0.00ns	2	0.20ns	1.10ns
W*N*Y	8	2.81*	2.2*	0.72ns	5	0.24ns	0.18ns

§ Pr>F;

***<0.001;

***<0.01;

***<0.05;

ns.: not significant.

Nitrogen effects on ANPP were influenced by water and the variation in precipitation among years (Table 1). Under dry years of the NP treatment in 2005, 2006, and 2009, ANPP did not differ among the nitrogen treatments, while ANPP increased significantly with nitrogen application in the moist years of 2007 and 2008 (Figure 2B). Furthermore, ANPP responses to nitrogen addition were much higher than expected in 2008 when natural precipitation was similar to the long-term average for most of the growing season (Figure 2B). Under SWP, however, ANPP increased significantly with increasing nitrogen inputs in all five years (Figure 2C). In the SWP treatment, ANPP increased on average 31% and 61% in the 25 and 50 kg ha^−1^ nitrogen additions, respectively, compared to the no nitrogen treatment. Inter-annual variations of ANPP (CV_ANPP_, %) within nitrogen levels were significantly higher in the NP treatment than in the SWP treatment. Nitrogen addition increased CV_ANPP_ in the NP, but not in the SWP treatment (Figure 2D).

**Figure 3:**
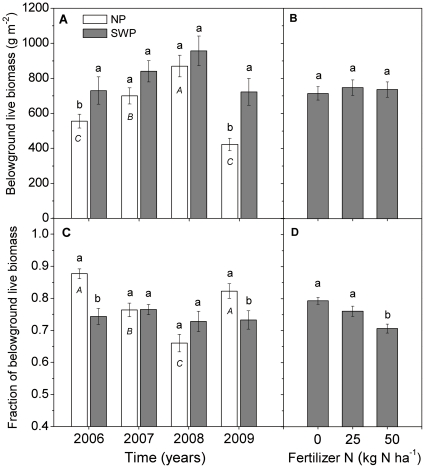
Belowground live biomass and the fraction of total dry mass allocated belowground. (A, C) Belowground live biomass (g m-2) and the fraction of belowground live biomass as affected by year and water interaction, with data pooled over all nitrogen treatments (n = 12). (B, D) Belowground live biomass and the fraction of belowground live biomass as influenced by nitrogen, with data pooled over all water treatments and years (n = 32). Bars labeled with the same lowercase letter were not significantly different between levels of water and nitrogen treatments (P>0.05). Bars labeled with the same italic capital letters were not significantly different among years in the NP treatment (P>0.05). There were no significant differences among years in the SWP treatment.

Only water availability, either across years or across watering treatments, influenced belowground live biomass. Belowground live biomass was higher in the moist years of 2007 and 2008 than in the dry years of 2006 and 2009 in the NP treatment (Figure 3A). On average, belowground live biomass was greater in the SWP treatment than in the NP treatment, especially in dry years (2006 and 2009) (Table 1, Figure 3A). Watering increased belowground biomass, but less than it increased aboveground biomass (Figure 2A and 3A). This influenced plant allocation, such that the ratio of biomass allocated belowground was lower in the SWP than in the NP treatment in dry years (2006 and 2009), but not in moist years (2007 and 2008, Figure 3C). Nitrogen application did not influence belowground live biomass (Table 1, Figure 3B), while the fraction of belowground live biomass decreased with increasing N inputs (Figure 3D) due to greater ANPP in fertilized treatments.

### Precipitation use efficiency and accumulated nitrogen fertilizer use efficiency

**Figure 4:**
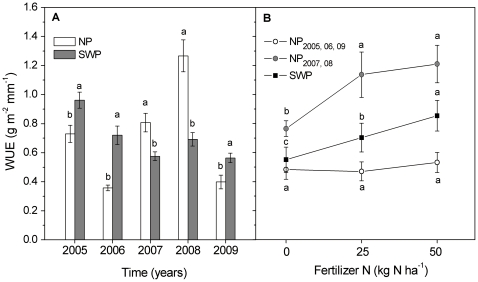
Water use efficiency (WUE, g m-2 mm-1) in response to water and nitrogen availability. (A) WUE as influenced by the year by water interaction, with data pooled over all nitrogen treatments (n = 12). Bars within years labeled with the same letter were not significantly different (P>0.05). (B) Nitrogen effect on WUE in NP treatment of dry years (2005, 2006, 2009, n = 12), moist years (2007 and 2008, n = 8) and SWP treatment (n = 20) respectively. Points within a line labeled with the same letter were not significantly different within water availability designations (P>0.05).

**Figure 5:**
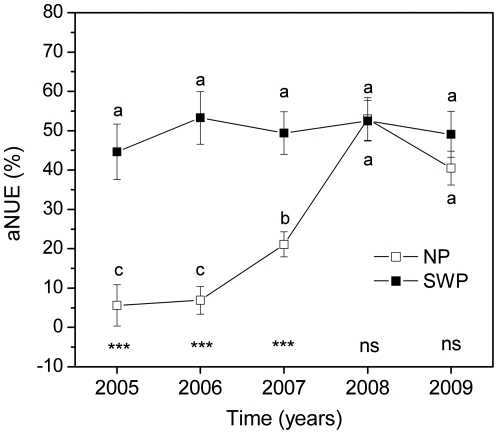
Accumulated nitrogen fertilizer use efficiency (aNUE, %) as affected by water over five years. Points labeled with the same letter were not significantly different among years (P>0.05). Effects of water are indicated by *** (P<0.001), ** (P <0.01), * (P <0.05) or ns (not significant). There were no significant differences between nitrogen treatments, so the values for N25 and N50 were pooled (n = 8).

Water use efficiency (WUE) varied among years, watering treatments, and fertilization treatments (Table 1). Water addition increased WUE in dry years (2005, 2006 and 2009), whereas it decreased WUE in moist years (2007, 2008) (Figure 4A). Nitrogen application significantly increased WUE in moist years of NP and in all years of the SWP treatment (Figure 4B). In concert with the lack of effect of nitrogen on ANPP in dry years of the NP treatment, nitrogen addition did not affect WUE in dry years (Figure 4B). Overall, the accumulated nitrogen fertilizer use efficiency (aNUE) was significantly higher in SWP than in NP (Figure 5). In the SWP treatment, aNUE was 50±3%, with no difference found among the five years (Figure 5). In the NP, however, aNUE increased 300–800% over the same time period, from only 6% in 2005 and 2006 to 53% in 2008 (Figure 5).

Added nitrogen accumulated in the upper soil layers in the NP treatments, but not in the SWP treatment (Table 2). The soil mineral nitrogen content was significantly higher in NP plots than in SWP plots at the beginning of the growing season before fertilization in 2007 (Table 2). Soil mineral nitrogen increased significantly with nitrogen fertilizer application in NP plots, while soil mineral nitrogen did not differ across fertilizer treatments in SWP plots (Table 2).

**Table 2:** Soil mineral nitrogen (kg N ha^−1^) before fertilization at the beginning of the 2007 growing season and results of two way ANOVAs to assess the effects of water (W) and nitrogen (N) on soil mineral nitrogen.

		Soil mineral nitrogen		
Precipitation	Nitrogen	0–15 cm	15–30 cm	30–50 cm
NP	N0	8.3±1.6 c	7.5±0.3 c	4.5±0.6 b
	N25	20±3.6 b	15±3.3 b	8.0±0.9 a
	N50	32±3.9 a	22±2.8 a	8.0±1.4 a
SWP	N0	11±1.2 a	3.8±0.8 a	3.5±0.5 a
	N25	14±2.8 a	4.7±1.2 a	3.5±0.8 a
	N50	8.5±1.5 a	5.9±0.8 a	4.0±0.8 a
Two way ANOVA		Probability of treatment effects		
	Water (W)	<0.001	<0.001	<0.001
	Nitrogen (N)	0.001	<0.001	0.069
	W*N	<0.001	0.006	0.129

Different small letters indicate significant differences among nitrogen levels within NP and SWP treatments and within soil layers (p<0.05, n = 4).

### Aboveground biomass of perennial and annual species

**Figure 6:**
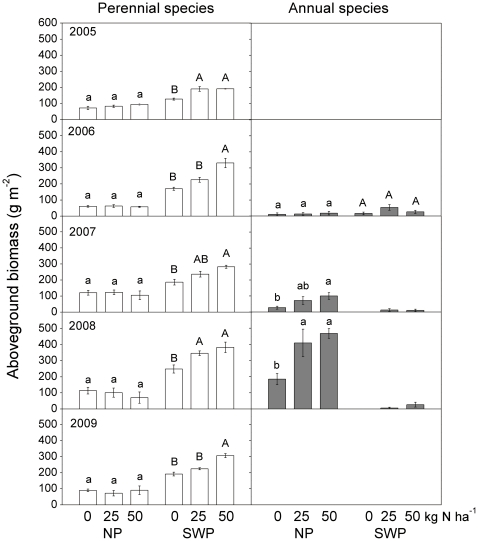
Aboveground biomass of perennial and annual species as affected by water and nitrogen supplementation. Bars within each water treatment labeled with the same letter were not significantly different (P>0.05, n = 4).

Differences across years in ANPP in the NP treatment were driven primarily by the responses of annual plants, rather than by perennial grasses, which are the primary forage species. During the whole experimental period, annual species were absent from or at very low abundances in the SWP plots at the HG site (Figure 6). In the NP plots, the relative abundance of annual species was lower than 16% in 2005 and 2006, but unpalatable annuals (*Salsola collina* and *Chenopodium glaucum*)suddenly dominated (47–87%) following nitrogen addition in the wet years of 2007 and 2008, before disappearing again in 2009. While aboveground biomass of annuals greatly increased with 25 kg ha^−1^ or 50 kg ha^−1^ nitrogen application in the NP treatment, aboveground biomass of perennial species did not (Figure 6). Aboveground biomass of perennial species was higher in the SWP than the NP treatment, and responded significantly to nitrogen addition in the SWP treatment as well (Figure 6).

## Discussion

Our results indicated that the strength of nutrient limitation in these degraded grasslands depended strongly on water availability. Similar to other studies [15], [36], we found the primary limiting factor for production shifted from water to nitrogen with increasing precipitation. The extent to which low levels of nitrogen fertilization could act as a tool to improve primary production in degraded grasslands depends on several issues. On one hand, the leftover fertilizer nitrogen in the soil from dry years remained available for uptake during wet years. Plant growth by residual fertilizer nitrogen could therefore lead to sufficient yields to offset lack of higher fertilized yields in dry years. However, the utility of this approach will depend on the influence of additional nitrogen on species composition, forage quality, and long-term sustainable stocking rates with increased inter-annual variation of ANPP under nitrogen addition [12], [37], [38].

### Shift of primary limiting factor on production from water to nitrogen

We found a clear shift from water to nitrogen limitation in response to inter-annual and experimental increases in water availability in this ecosystem. In dry years, precipitation was the primary factor limiting the production of the Inner Mongolian grassland (Figure 2 and 4A). We saw nitrogen limitation in 2007, which was a lower precipitation year but with even precipitation distribution. Finally, when precipitation was similar to the long-term average (NP in 2008) and reached the mean value of wet year precipitation (SWP), the primary limiting factor for ANPP shifted from water to nitrogen (Figure 2). For this reason, ANPP cannot be predicted only by precipitation amount, particularly in wet years [21], [39].

Water and nitrogen fertilizer use efficiencies also reflected the shift in limiting factors with changing precipitation. In our study, water addition significantly increased WUE in dry years, but decreased WUE in moist years (2007, 2008; Figure 4A). This was consistent with the findings of other studies that WUE was low at both dry and wet extremes of the annual precipitation range and that peak WUE occurred at moderate precipitation levels [40], [41]. Several mechanisms could explain this transition in WUE. In water-limited environments, plants adapt with traits such as small plant size, low specific leaf area, slow growth, and low rates of tissue turnover, leading to trade-offs between relative growth rate and drought resistance [42], [43]. In very dry years, plant survival may restrict ANPP and WUE. In moist years, water addition likely decreased WUE due to limited growth response from functional trait tradeoffs and the shift to nitrogen limitation at high water availability (Figure 4B) [22], [44].

Nitrogen fertilizer use efficiency was affected by water availability. When water availability was consistently high, accumulated nitrogen fertilizer use efficiency was relatively stable (50±3%, SWP treatments), and was much higher than that of low water availability treatments (25±21%, averaged across all NP treatments and years). The large increase in aNUE from the dry years of 2005 and 2006 to the moist years of 2007 and 2008 is particularly striking because the additional growth and nitrogen uptake responses in 2007 and 2008 were strong enough to offset the low production in the previous two years (Figure 5). The nitrogen left in the soil from fertilization in previous dry years (Table 2) remained available for uptake during wet years, helping to fuel this additional growth. This pattern suggests that plant growth by residual fertilizer nitrogen could lead to sufficient yields to offset lack of increased yields from fertilizer in dry years.

Different ecosystems with different climate conditions and grassland management may respond to additional nitrogen in different ways [30] and differences in the precipitation threshold from water to nitrogen limitation can have important implications for management. Research in California grasslands indicated co-limitation of water and nitrogen on production in a year of below average rainfall [45]. In contrast, in savanna ecosystems in Africa, water was the primary limiting factor for productivity, with no effect of nitrogen addition on ANPP until precipitation was at least 130% of the average [46]. In this experiment in Inner Mongolian steppe, nitrogen limitation on production occurred when precipitation was similar to the long-term average year (NP in 2008) or even in a lower precipitation year with good precipitation distribution (2007, Figure 1 and 2B), indicating the possibility that nitrogen fertilizer could serve as a tool for restoring degraded grasslands in that region.

Despite the seemingly promising response of production to residual fertilizer nitrogen, however, almost all of the additional growth in wet years in the NP treatment resulted from annual species with low palatability. These species became dominant in moist years (2007 and 2008) and then disappeared in the following dry year (2009, Figure 6). Bai et al. (2008) also found higher WUE with fertilization that resulted primarily from increased growth of annuals [36], as in this study. The low palatability and unpredictability of this growth may constrain stocking rates to those sustainable in drier years in heavily grazed sites [47], [48]. This poses challenges for herders who seek to maximize their revenue in any given year but who also need to avoid overgrazing in dry years. Future studies are needed that focus on how plant functional traits relevant to grazing are affected by resource addition and precipitation variation.

In parallel with the responses of ANPP, belowground production improved significantly with increasing water availability (Figure 3A). Water addition (SWP treatment) led to consistent allocation to roots (∼25%) and shoots (∼75%) across all years, which was similar to allocation in moist years in NP (Figure 3C). Fractional root allocation in dry years (2006 and 2009) was high, however, and greatly decreased with water input (Figure 3C). These results support the functional equilibrium theory [49]. According to this idea, shoot and root growth co-ordinate to balance the water demand of leaf transpiration with the water absorbing capacity of roots. Belowground resource limitations (such as water or nitrogen shortage) promote dry mass allocation to belowground parts [50], [51], while aboveground resource (light) limitation promotes more dry mass allocation to shoots [52]. Nitrogen addition did not change live belowground biomass but greatly decreased proportional root allocation (Figure 3B, D), presumably to compete for light [53]. Similar findings occurred in tallgrass prairie ecosystems in North America [54]. Unlike the ANPP response to nitrogen addition, belowground production did not respond to nitrogen fertilizer in these degraded grasslands, suggesting little additional carbon storage or organic matter accumulation from roots with low levels of nitrogen fertilization. The strength of this conclusion depends on rates of root and soil organic carbon turnover, however.

### Response of overgrazed grasslands to stable and unstable resource availability

Variability in ANPP was influenced by a combination of precipitation variation and biogeochemical constraints such as nitrogen availability [40], [55]. Under the conditions of simulated wet year precipitation (SWP), with similar amount and pattern of precipitation across the 5 years, inter-annual variation of ANPP (CV_ANPP_) was low and there was no nitrogen effect on CV_ANPP_ (Figure 1 and Figure 2D). However, strong variation of ANPP was found among years with highly variable precipitation (Figure 1, Figure 2B, D), and CV_ANPP_ increased even more with nitrogen addition. Low nitrogen availability, on the one hand, is one of the most important limiting factors for production in Inner Mongolian grasslands [12]; on the other hand, the extent of nitrogen limitation depended heavily on water availability (Figure 2B, C). Due to strong inter-annual variation in precipitation in this ecosystem, there might be a high frequency of transition between water and nitrogen as limiting factors for ANPP [22] (Figure 2B).

The question remains unresolved as to whether application of small amounts of nitrogen fertilizer can serve as a tool for grassland restoration for heavy grazing. A four-year nitrogen fertilizer experiment in this ecosystem indicated that nitrogen addition increased ANPP, and that the dominance of perennial rhizomatous species was important for the restoration of degraded grasslands [12]. However, that study did not evaluate the interactive effects of nitrogen and precipitation on production, and the precipitation during that experiment period was stable and close to the long-term average precipitation (crop year precipitation was 309±29mm). In contrast, precipitation during our study period was even lower than long-term mean dry years, with crop year precipitation varying from 190 to 354 mm (Figure 1). Several global climate change models have predicted that between-year variability in precipitation will increase in the future [16], [56], [57]. Facing such variability, combined with the frequent shift from water to nitrogen limitation, our five year results suggested plant growth by residual fertilizer N in dry years increased yields in following wet years. However, most of this increase resulted from unpalatable annual forbs, with no effect on more desirable perennial grasses. Interestingly, these annual species disappeared again in a subsequent dry year. Considering these effects of nitrogen application, longer-term study is needed to comprehensively appraise the impact of low-level nitrogen addition on ecosystem stability and the possible change of plant species composition in Inner Mongolian grasslands.

## Supporting Information

Figure S1 **Average ten day temperature (oC) among experiment years.**
(TIF)

Figure S2 **Average soil moisture from the beginning and the end of growing seasons at 0-30 cm soil depth.** Data were averaged across all nitrogen treatments (n = 12).
(TIF)
