# Supplementary figures and images for: Shifts in Species Composition Constrain Restoration of Overgrazed Grassland Using Nitrogen Fertilization in Inner Mongolian Steppe, China
Source: PLoS One. 2011 Mar 1;6(3):e16909. doi: 10.1371/journal.pone.0016909 (PMC3046964; doi:10.1371/journal.pone.0016909)

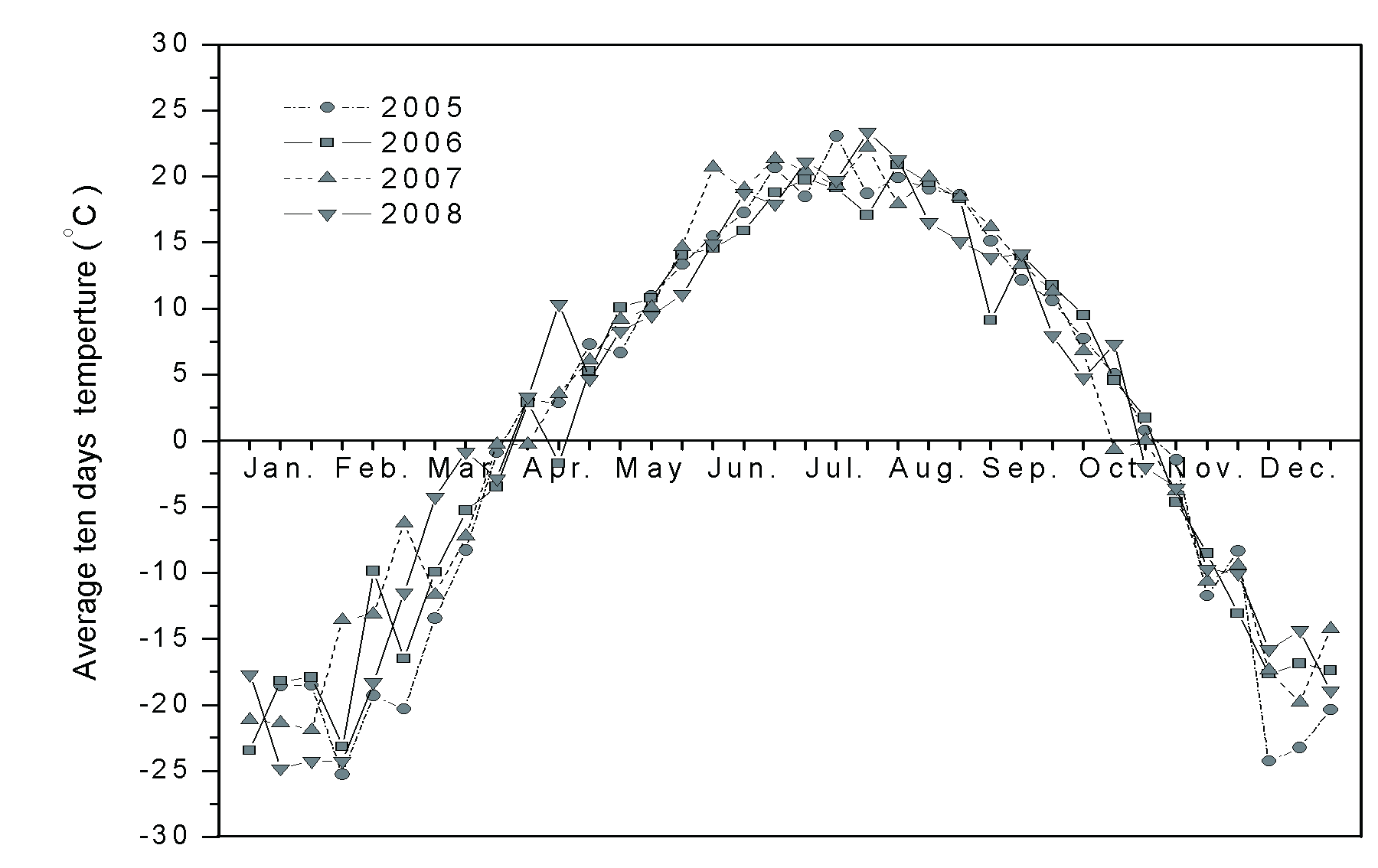

Supplement: Figure S1 — Average ten day temperature (oC) among experiment years. (TIF) [file pone.0016909.s001.tif]

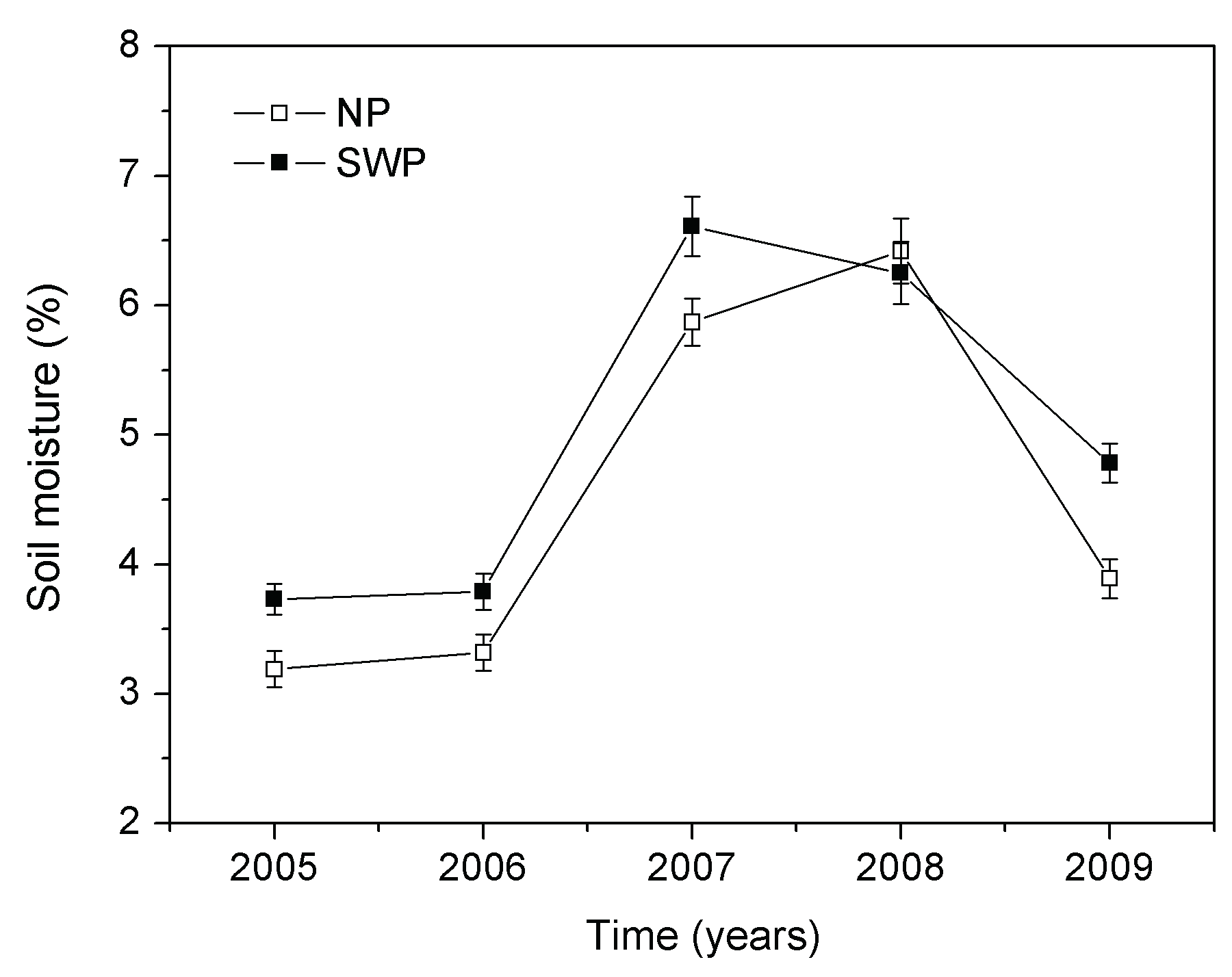

Supplement: Figure S2 — Average soil moisture from the beginning and the end of growing seasons at 0-30 cm soil depth. Data were averaged across all nitrogen treatments (n = 12). (TIF) [file pone.0016909.s002.tif]
